# Mineralocorticoid receptor antagonist pre-treatment and early post-treatment to minimize reperfusion injury after ST-elevation myocardial infarction: The MINIMIZE STEMI trial

**DOI:** 10.1016/j.ahj.2019.02.005

**Published:** 2019-05

**Authors:** Heerajnarain Bulluck, Georg M Fröhlich, Jennifer M Nicholas, Shah Mohdnazri, Reto Gamma, John Davies, Alex Sirker, Anthony Mathur, Daniel Blackman, Pankaj Garg, James C Moon, John P Greenwood, Derek J Hausenloy

**Affiliations:** a Hatter Cardiovascular Institute, London, United Kingdom; b Barts Heart Centre, St Bartholomew's Hospital, London, United Kingdom; c Charité - Universitätsmedizin Berlin, Germany; d London School of Hygiene and Tropical Medicine, London, United Kingdom; e Essex Cardiothoracic Centre, Basildon, United Kingdom; f Leeds Institute of Cardiovascular and Metabolic Medicine, University of Leeds, Leeds, United Kingdom; g The National Institute of Health Research University College London Hospitals Biomedical Research Centre, London, United Kingdom; h National Heart Research Institute Singapore, National Heart Centre Singapore, Singapore; i Cardiovascular and Metabolic Disorders Program, Duke-National University of Singapore, Singapore; j Yong Loo Lin School of Medicine, National University Singapore, Singapore; k Tecnologico de Monterrey, Centro de Biotecnologia-FEMSA, Nuevo Leon, Mexico

## Abstract

**Background:**

Mineralocorticoid receptor antagonist (MRA) therapy has been shown to prevent adverse left ventricular (LV) remodeling in ST-segment elevation myocardial infarction (STEMI) patients with heart failure. Whether initiating MRA therapy prior to primary percutaneous coronary intervention (PPCI) accrues additional benefit of reducing myocardial infarct size and preventing adverse LV remodeling is not known. We aimed to investigate whether MRA therapy initiated prior to reperfusion reduces myocardial infarct (MI) size and prevents adverse LV remodeling in STEMI patients.

**Methods:**

STEMI patients presenting within 12 hours and with a proximal coronary artery occlusion with Thrombolysis In Myocardial Infarction flow grade 0 were consented and randomized to either an intravenous bolus of potassium canrenoate, followed by oral spironolactone for 3 months or matching placebo. The primary endpoint was MI size by cardiovascular magnetic resonance at 3 months.

**Results:**

Sixty-seven patients completed the study. There was no significant difference in the final MI size at 3 months between the 2 groups (placebo: 17 ± 11%, MRA: 16 ± 10%, *P* = .574). There was also no difference in acute MI size (26 ± 16% versus 23 ± 14%, *P* = .425) or myocardial salvage (26 ± 12% versus 24 ± 8%, *P* = .456). At follow-up, there was a trend towards an improvement in LVEF (placebo: 49 ± 8%, MRA: 54 ± 11%, *P* = .053), and the MRA group had significantly greater percentage decrease in LVEDV (mean difference: −12.2 (95% CI −20.3 to −4.4)%, *P* = .003) and LVESV (mean difference: −18.2 (95% CI −30.1 to −6.3)%, *P* = .003).

**Conclusion:**

This pilot study showed no benefit of MRA therapy in reducing MI size in STEMI patients when initiated prior to reperfusion, but there was an improvement in LV remodeling at 3 months. Adequately powered studies are warranted to confirm these findings.

Mortality in patients with acute ST-segment elevation myocardial infarction (STEMI) has declined over the past 4 decades^1^ but morbidity due to post-myocardial infarction (MI) heart failure, risks for arrhythmias and repeat ischemic events remains significant.^2^ The process of reperfusion itself can paradoxically induce further myocardial injury and cardiomyocyte death as a consequence of ‘myocardial reperfusion injury’^3^ and the latter can contribute up to 50% of the final MI size.^4^ Despite a wealth of research in this field, there is currently no effective therapy for reducing myocardial reperfusion injury.^5^ This has been partly attributed to the unfavorable timing and mode of delivery of the cardioprotective agent; poor selection of patients; and suboptimal choice of endpoints.^5^

Pre-clinical data in murine, rat and rabbit *in vivo* models of MI have demonstrated that administering either intravenous potassium canrenoate (a compatible metabolite of spironolactone) or eplerenone after a sustained episode of myocardial ischemia and 5 minutes prior to reperfusion, protected the heart against myocardial reperfusion injury and reduced MI size by 40–50%.^6^

Therefore, the MINIMIZE STEMI trial^7^ was designed to assess the benefit of mineralocorticoid receptor antagonist (MRA) therapy in STEMI patients without heart failure on reducing MI size and preventing adverse left ventricular (LV) remodeling. We hypothesized that early intravenous MRA therapy administered prior to restoration of flow in the infarct-related artery, followed by 3 months oral MRA therapy could reduce MI size and improve LV remodeling in STEMI patients.

## Methods

### Study population

The MINIMIZE-STEMI trial (https://clinicaltrials.gov, NCT01882179) was a prospective, proof-of-concept, multi-center, double-blinded randomized placebo controlled clinical trial.^7^ The study was conducted in accordance with the Declaration of Helsinki and was approved by the UK National Research Ethics Service. All patients provided written informed consent. Consecutive STEMI patients were screened from 4 centers in the United Kingdom between December 2013 and January 2016. The study design has been previously described.^7^

In brief, the main inclusion criteria were patients >18 years, with an acute STEMI (as assessed by 12 lead ECG; ST segment elevation ≥2 mm (0.2 mV) in 2 or more contiguous precordial leads or ≥1 mm (0.1 mm) in 2 or more adjacent limb leads), presenting within 12 hours of symptoms onset. The angiography inclusion criteria were TIMI 0 in a proximal left anterior descending, circumflex or right coronary artery territory STEMI and the initial serum potassium of <5.0 mmol/l. Patients with known previous MI, heart failure or LVEF ≤40%, in cardiogenic shock, estimated glomerular filtration rate <30 mL/min per 1.73 m^2^, unable to consent, on pre-existing MRA therapy or with known contraindication to cardiovascular magnetic resonance (CMR) imaging were excluded.

### Study protocol

On immediate arrival at the primary percutaneous coronary intervention (PPCI) center, eligible patients were consented to enter the MINIMIZE STEMI trial. Patients were randomized via a web-based service (www.SealedEnvelope.com) in a 1:1 manner to either MRA therapy or matching placebo. Randomization was stratified by recruiting site. The study drug or placebo was administered by the unblinded research investigator. The patient, PPCI operator, and research staff collecting the data were blinded to the treatment allocation.

#### MRA therapy

Patients randomized to MRA therapy received an intravenous bolus of 200 mg (10 ml) of potassium canrenoate^8^ prior to restoration of flow in the infarct-related artery, followed by oral spironolactone 25 mg once daily for 2 weeks and then 50 mg once daily (if serum potassium level allowed) for the remaining 10 weeks. Renal function was assessed at 2 and 4 weeks.

#### Placebo

Patients randomized to placebo received a 10 ml intravenous bolus of normal saline followed by placebo tablets for the 3 months.

Patients with an LVEF ≤40% on the initial CMR scan and evidence of heart failure or who were diabetic were started on open-label eplerenone according to current practice guidelines. These patients were included in the intention-to-treat analysis. A CMR was performed within the first week following PPCI and was repeated 3 months later. A pre-specified interim analysis was planned on safety grounds.

The primary endpoint was MI size by CMR at 3 months. Pre-specified secondary endpoints included acute MI size by CMR, extent and incidence of microvascular obstruction (MVO) on CMR, myocardial salvage and indices of LV remodeling (end-diastolic volume (LVEDV), end-systolic volume (LVESV) and LVEF.

In addition to the above analysis of pre-specified outcomes, two post-hoc outcomes were examined: percentage change in LVEDV and percentage change in LVESV. These were defined using the equation below:

$$\mathrm{Volume}\;\mathrm{change}\left(\%\right)=\left({\mathrm{Volume}}_{3\;\mathrm{months}}–{\mathrm{Volume}}_{1\;\mathrm{week}}\right)/{\mathrm{volume}}_{1\;\mathrm{week}}\;x\;100$$

### CMR acquisition and analysis

The CMR protocol has been previously described.^7^ In brief, all CMR scans were performed on a 1.5 Tesla scanner. Full short axis LV coverage cine images (for volumes, LVEF and mass), T2-mapping or T2-weighted imaging (for the edema-based area-at-risk) and LGE imaging (10 minutes after 0.1 mmol/kg of Gadoterate meglumine for MI size) were acquired. Matching sequences with the same parameters were used at follow-up.

All imaging analysis was performed using CVI42 software (Version 5.1.2[303], Calgary, Canada). The endocardial and epicardial borders were manually delineated on all the cine, T2 and LGE short-axis LV images. LVEF, LV volumes and LV mass were quantified using the summation of discs method.^9^ A reference region of interest was drawn in the remote normal myocardium. The area-at-risk and MI size were quantified using a signal intensity threshold of 2 and 5 standard deviations (SD) above the normal remote myocardium, respectively.10., 11. Areas of hypo-intense core of MVO were included as part of the MI zone and edema-based area-at-risk.

Myocardial salvage was calculated as area-at-risk by T2 on the first CMR minus the MI size at 3 months and was expressed as a percentage of the LV.

### Sample size calculation

The study was originally powered for a sample size of 50 in each group, to give 80% power to detect a difference in means of 8.0 g infarct mass by CMR assuming that the common standard deviation was 14.0 g.7., 12. Accounting for dropouts, a total sample size of 150 patients was planned. An interim analysis was performed after 70 patients were recruited.

### Statistical analysis

All analysis was performed using Stata version 15.0 (StataCorp, College Station, TX). MI size was compared between the 2 groups using linear regression with the MI size as the response variable and the treatment group included as a binary covariate in the model. The distribution of both acute and follow-up MI size in grams showed marked positive skew, but this distribution was normalized by expressing MI size as percentage of the LV and the latter was used for analysis. The extent of MVO was compared between groups using linear regression. Due to non-normal distribution of the outcome, we used non-parametric bias-corrected and accelerated bootstrapped 95% confidence intervals based on 2000 replications stratified by treatment group. Myocardial salvage was expressed as a percentage of LV and compared using linear regression. Linear regression was also used to compare the groups on LVEDV, LVESV, LVEF, and LV mass at 3 months. Analysis of LV mass used robust standard errors to allow for heteroscedasticity in this measure.

Due to observed baseline imbalances between the groups in participants with clinical data available, we conducted a post-hoc secondary adjusted analysis of the primary and secondary endpoints. This analysis adjusted for age, history of dyslipidemia, infarct-related artery, and duration of ischemia by including these as predictors in the linear regression model along with treatment group.

## Results

### Patient recruitment

**Figure 1:**
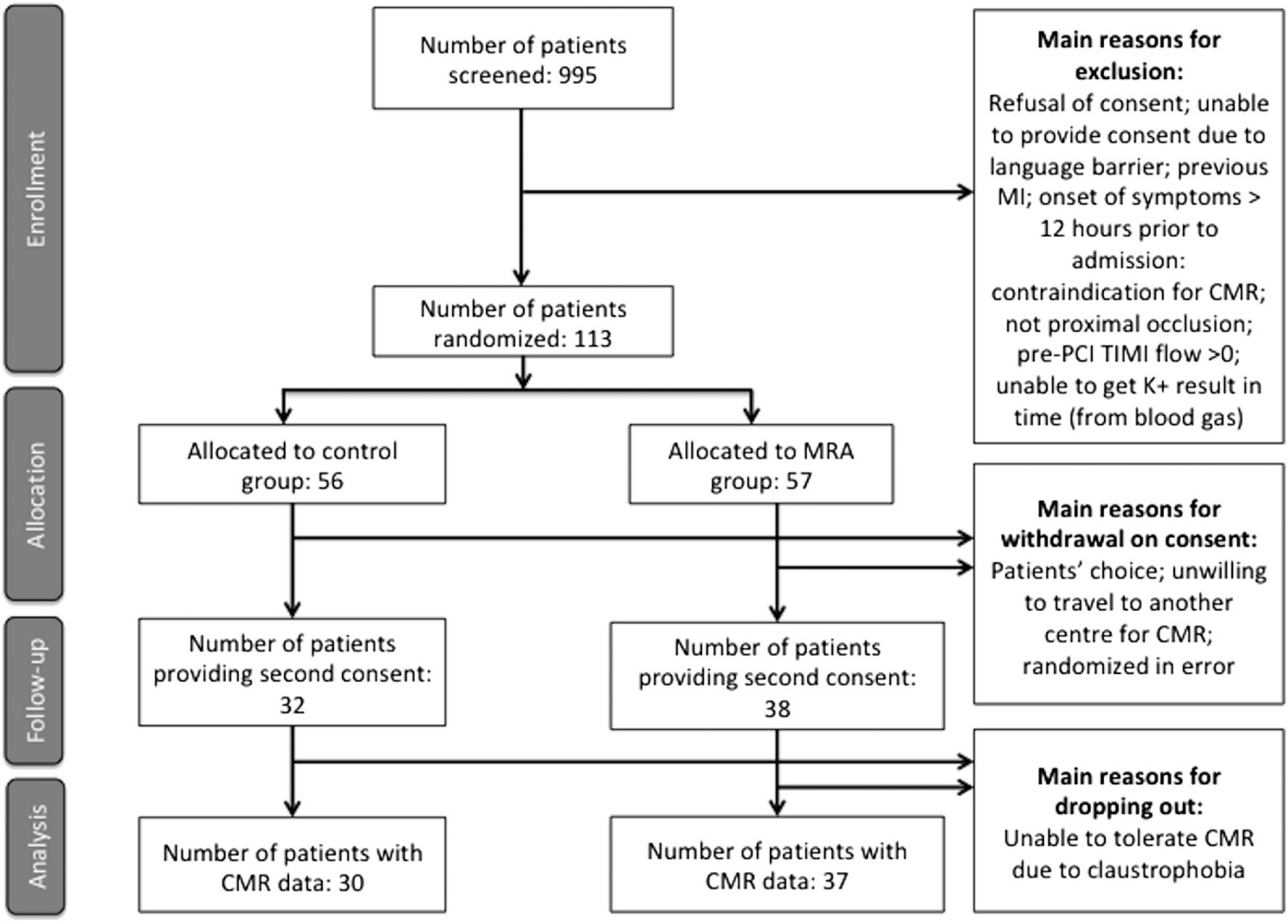
CONSORT diagram for the MINIMIZE STEMI trial. This is a summary of the patient selection, recruitment and follow-up.

Nine hundred ninety-five patients were screened from the 4 centers over the 2-year period. Figure 1 shows the CONSORT diagram for this study. There were 113 patients randomized at the time of PPCI following brief informed consent and 70 patients agreed to stay in the study after the second comprehensive informed consent. CMR data were available in 67 patients.

### Baseline characteristics

Among the 70 patients with clinical data available, there were some imbalances in the baseline characteristics between the MRA group and placebo group. Those in the MRA group were more likely to have higher blood pressure and higher heart rate on admission, and were more likely to be smokers (Table I). Those in the MRA group were less likely to have a history of dyslipidemia (29% vs 47%), but were more likely to have a family history of coronary artery disease (53% vs 41%) than those in the placebo group (Table I). Medication use at baseline was broadly similar in the two groups. The infarct-related artery was most likely to be the right coronary artery in the MRA group but the left anterior descending artery in the placebo group. Duration of ischemia and call to door time were both more likely to be longer in the MRA group than in the placebo group (Table I). These imbalances motivated the secondary post-hoc analysis, as described above, which adjusted for the potentially important prognostic characteristics that appeared to show an imbalance between the two groups.

**Table I:** Baseline characteristics of the patients

	Placebo (n = 32)	MRA (n = 38)	*P*
Age, mean ± SD (years)	60 ± 13	62 ± 10	.47
Male gender (%)	27 (84)	33 (87)	1.0
Smoking status, N (%)			.57
Non-smoker	12 (38)	15 (39)	
Ex-smoker	11 (34)	9 (24)	
Current smoker	9 (28)	14 (37)	
Prior diagnoses, N (%)			
Hypertension⁎	11 (34)	13 (35)	1.0
Dyslipidemia	15 (47)	11 (29)	.14
Diabetes Mellitus	4 (13)	2 (5)	.40
Stroke or TIA	2 (6)	0 (0)	.21
PVD	0 (0)	1 (3)	1.00
COPD	2 (6)	9 (24)	.06
BMI, mean ± SD (kg/m^2^)⁎	28 ± 4	28 ± 4	.47
SBP, mean ± SD (mmHg)	126 ± 22	135 ± 31	.17
DBP, mean ± SD (mmHg)	78 ± 17	88 ± 24	.06
HR, mean ± SD (beats/min)	72 ± 16	75 ± 18	.47
Pre-existing medications, N (%)			
Beta-blockers	4 (13)	3 (8)	.70
ACEI/ ARB	3 (9)	5 (13)	.68
Statins	7 (22)	8 (21)	1.00
Infarct-related artery, N (%)			.06
LAD (%)	19 (59)	15 (39)	
LCX (%)	3 (9)	1 (3)	
RCA (%)	10 (31)	22 (58)	
Call to balloon time, median (IQR) (minutes)	144 (115 to 191)	173 (114 to 245)	.35
Door to balloon time, median (IQR) (minutes)⁎	31 (27 to 44)	39 (30 to 53)	.29
Radial access (%)⁎	25 (78)	31 (84)	.55
DES use (%)	29 (91)	36 (95)	.65
Heparin use (%)	31 (97)	37 (97)	.20
Bivalirudin (%)	4 (13)	4 (11)	1.0
Aspirin (%)	30 (94)	36 (95)	.63
Clopidogrel (%)	7 (22)	8 (21)	.58
Prasugrel (%)	6 (19%)	10 (26%)	.32
Ticagrelor (%)	19 (59)	19 (50%)	.29

Abbreviations: *SD*, Standard deviation; *TIA*, transient ischemic attack; *PVD*, peripheral vascular disease; *COPD*, chronic obstructive pulmonary disease; *BMI*, body mass index; *SBP*, systolic blood pressure; *DBP*, diastolic blood pressure; *HR*, heart rate; *ACEI*: angiotensin-converting enzyme inhibitor; *ARB*: angiotensin receptor blocker; *LAD*, left anterior descending artery; *LCX*, left circumflex artery; *RCA*, right coronary artery; *IQR*, inter-quartile range; *DES*, drug-eluting stent.

⁎ These characteristics were recorded for the following numbers of patients: hypertension, door to balloon time, and radial access N = 69; BMI N = 68. All other characteristics were recorded for N = 70 patients.

One patient in the MRA group died during the follow-up period and 2 patients from the same group had mild and transient episodes of hyperkalemia.

### Primary and secondary endpoints

Three patients in the MRA group and 1 patient in the placebo group were discharged on open-label MRA therapy (eplerenone) as per current guidelines.^13^,^14^

## Intention-to-treat analysis

**Figure 2:**
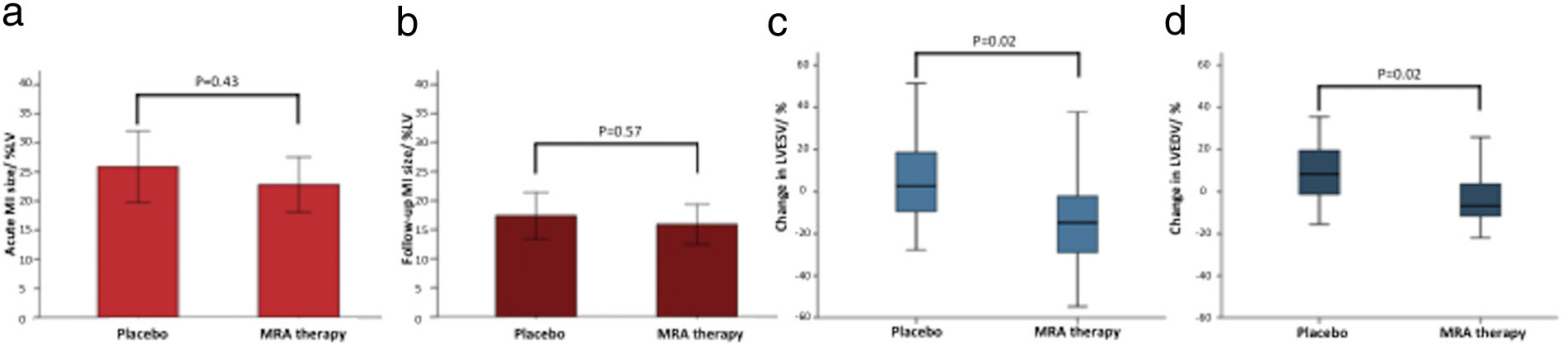
Bar charts of acute (a) and chronic (b) MI size and box and whisker plots of percentage change in LVESV (c) and LVEDV (d) in the placebo and MRA therapy group. There was no significant difference in both (a) acute and (b) chronic MI size as shown in the bar charts. There was significant great percentage reduction in (c) LVEDV and (d) LVESV in the MRA group as shown in the box and whisker plots. *Bar charts: box representing mean and error bars represent ± 2 × standard error.* *Box and whisker plot: box representing median and interquartile range and whiskers representing maximum and minimum*.

There was no significant difference in the primary endpoint of MI size at 3 months between the 2 groups (placebo group: mean ± SD 17 ± 11%, MRA group: 16 ± 10%, *P* = .574, Figure 2b). There was also no difference in acute MI size (placebo group: 26 ± 16%, MRA group: 23 ± 14%, *P* = .425, Figure 2a) or myocardial salvage (placebo group: 26 ± 12%, MRA group: 24 ± 8%, *P* = .456) between the 2 groups.

The other CMR parameters between the 2 groups are provided in Table II. There was no difference in the extent of MVO between the 2 groups. However, there was a significantly greater percentage decrease in LVESV in the MRA therapy group compared to placebo (mean difference: −18.2 (95%CI −30.1 to −6.3)%, *P* = .003) and also significant evidence for more favorable change in LVEDV (mean difference: −12.2 (95% CI −20.3 to −4.4)%, P = .003) (Table II and Figure 2c and d). There was no difference in the baseline LVEF between the 2 groups (placebo group: 46 ± 9%, MRA group: 48 ± 9%, *P* = .283). There was a trend towards an improvement in LVEF at follow-up but this did not reach statistical significance (placebo group: 49 ± 8%, MRA group: 54 ± 11%, *P* = .053).

**Table II:** Primary analysis and secondary post hoc adjusted analysis of the primary and secondary outcomes

Outcome	Placebo		MRA		Primary analysis			Secondary post-hoc adjusted analysis		
	N	Mean ± SD	N	Mean ± SD	Difference in means⁎	95% CI	*P*	Difference in means⁎⁎	95% CI	*P*
Acute CMR										
MI size (%LV)	27	26 ± 16	34	23 ± 14	−3.1	−10.7 to 4.6	.425	0.8	−6.4 to 8.0	.819
MVO (g)⁎⁎⁎	27	5 ± 9	34	4 ± 8	−0.6	−6.0 to 3.0		0.4	−3.6 to 3.3	
Follow-up CMR										
MI size (%LV)	30	17 ± 11	32	16 ± 10	−1.5	−6.8 to 3.8	.574	0.6	−4.2 to 5.4	.795
EDV (ml)	30	188 ± 52	32	171 ± 46	−17.1	−41.9 to 7.7	.174	0.3	−23.8 to 24.4	.979
ESV (ml)	30	99 ± 40	32	82 ± 37	−16.2	−35.8 to 3.4	.104	−2.4	−21.0 to 16.2	.799
LV mass (g)	30	116 ± 21	32	112 ± 25	−3.7	−15.6 to 8.1	.531	0.4	−12.1 to 13.0	.945
LVEF (%)	30	49 ± 8	32	54 ± 11	4.8	−0.1 to 9.7	.053	1.9	−2.8 to 6.7	.412
Acute + follow-up CMR										
Myocardial salvage (%LV)	28	26 ± 12	32	24 ± 8	−1.9	−7.0 to 3.2	.456	1.1	−4.1 to 6.3	.662
Change in LVEDV (%)	27	10 ± 14	31	−3 ± 16	−12.3	−20.3 to −4.4	.003	−9.3	−17.9 to −0.6	.036
Change in LVESV (%)	27	6 ± 20	31	−12 ± 24	−18.2	−30.1 to −6.3	.003	−16.9	−29.9 to −3.8	.012

Abbreviations: *SD*, Standard deviation; *CI*, confidence interval; *MI*, myocardial infarction; *MVO*, microvascular obstruction; *LV*, left ventricle; *LVEDV*, LV end-diastolic volume; *LVESV*, LV end-systolic volume; *LVEF*, LV ejection fraction.

⁎ Difference estimated from a simple linear regression model with a binary indicator variable for treatment group.

⁎⁎ Difference estimated from a linear regression model with a binary indicator variable for treatment group, and adjusting for age, history of dyslipidemia (high cholesterol), infarct-related artery, and duration of ischemia.

⁎⁎⁎ Due to non-normal distribution of this outcome, inference was based on 95% CI from bias corrected accelerated bootstrapping with 2000 replications and no *P* value can be provided.

## Per-protocol analysis

There was no significant difference in the primary endpoint of MI size at 3 months between the 2 groups (placebo group: 17 ± 11%, MRA group: 14 ± 9%, *P* = .288). There was also no difference in acute MI size (placebo group: 26 ± 16%, MRA group: 21 ± 12%, *P* = .195) or myocardial salvage (placebo group: 26 ± 12%, MRA group: 23 ± 8%, *P* = .418) between the 2 groups. However, the MRA group had significantly higher LVEF at follow-up when compared to the placebo group (55 ± 10% versus 49 ± 8%, *P* = .016) and significantly greater percentage decrease in LVESV and LVEDV (*P* < .001 for both).

### Adjusted post-hoc analysis

The results of the post-hoc secondary analysis, adjusted for age, history of dyslipidemia, infarct-related artery, and duration of ischemia are shown in Table II and they were very similar to the unadjusted analysis.

There was no evidence of a difference between the placebo group and MRA group on MI size at 3 months post-PPCI. After adjustment, the mean follow-up MI size was 0.6 percentage points higher in the MRA group compared to placebo (95% CI −4.2 to 5.4; *P* = .80). There was also no evidence of a difference between the placebo group and MRA group for MVO, myocardial salvage, acute MI size and pre-specified measures of LV remodeling (Table II). However, those randomized to the MRA group had a significantly greater percentage decrease in LVEDV and LVESV between the follow-up and acute CMR (Table II).

## Discussion

This pilot study showed that MRA therapy initiated prior to reperfusion and continued for 3 months did not reduce reperfusion injury in STEMI patients treated by PPCI, when compared to placebo. However, those receiving MRA therapy showed less adverse LV remodeling at follow-up.

In the pre-clinical setting, intravenous potassium canrenoate (a compatible metabolite of spironolactone) or eplerenone have been shown to protect the heart against myocardial reperfusion injury and to reduce MI size significantly in rats, mice and rabbits.^6^ However, van den Berg et al.^15^ recently showed that eplerenone did not reduce ischemia–reperfusion injury in an ex-vivo model of human atrial tissue obtained from 24 patients undergoing cardiac surgery. Therefore our study was important, as it was the first study to assess whether MRA therapy administered prior to reperfusion provided a benefit against reperfusion injury and led to smaller MI size in the clinical setting. Furthermore, the patients also had 3 months of oral MRA therapy to assess whether our approach led to better post-MI LV remodeling at 3 months in those not meeting the criteria for MRA therapy.

MRA therapy has already been shown to prevent adverse LV remodeling following acute MI as summarized in a previous meta-analysis,^16^ and the findings from our study are consistent with this. The role of MRA therapy in acute MI patients with LVEF ≤40% and with signs and symptoms of heart failure or diabetes is well established.13., 14.^17^ However, the impact of MRA therapy on clinical outcomes in acute MI patients without heart failure is less well established. The REMINDER trial investigated the effect of initiating oral eplerenone therapy 12–24 hours following STEMI admission (in the absence of heart failure) in 1012 acute STEMI patients.^18^ There was a reduction in their composite endpoint in favor of eplerenone but this was mainly driven by lower brain natriuretic peptide levels. The ALBATROSS trial investigated the effect of MRA therapy (IV bolus of potassium canrenoate (200 mg) followed by a daily 25-mg dose of spironolactone for 6 months) initiated within 72 hours of symptom onset in 1603 patients with either NSTEMI or STEMI (regardless of heart failure status) on the 6 month primary combined endpoints.^19^ They failed to show the benefit of early MRA use in addition to standard therapy in that study. However, an exploratory analysis showed that there was a mortality benefit in the STEMI subgroup among those randomized to MRA therapy.^19^ A recent pooled analysis from the ALBATROSS and REMINDER trials reported that there were significantly fewer deaths in the MRA-treated patients when compared to controls after a median of 6 months follow-up.^20^ Two large meta-analyses recently showed that the mortality benefit was mainly in those patients with post-MI heart failure^21^ as well as in STEMI patients without heart failure.^22^ The beneficial effects of MRA therapy on mortality could be partly due to less adverse LV remodeling, a pre-cursor of heart failure and our study provides further support for the use of MRA therapy in STEMI patients. The mechanisms whereby MRA therapy contribute to less adverse LV remodeling is likely due to blockade of aldosterone-mediated increase in collagen synthesis^23^ and a reduction in extracellular matrix turnover.^24^

On the other hand, in a randomized study of 100 patients with acute MI and LVEF<40%, Weir et al.^25^ previously showed that eplerenone provided minimal incremental benefit against adverse LV remodeling over a 6 months period and these findings were attributed to the use of optimal contemporary pharmacological therapies. Of note, although they aimed to recruit patients with LVEF<40%, the LVEF by CMR was 51% when compared to 35% by screening echocardiography in the MRA group. It may have been the relatively higher baseline LVEF in the MRA therapy group in their study^25^ when compared to a mean LVEF of 48% on the baseline scan in our group that could have accounted for their findings.

The results from the pooled studies^20^ and meta-analyses21., 22. and our study support the need for an adequately powered study and the CLEAR-SYNERGY (NCT03048825) study (4000 patients; primary endpoint: composite of cardiovascular death or new or worsening heart failure at 2 years) has been designed to address this and is currently recruiting patients.

### Limitations

The inclusion criteria were strict but the study was designed to identify those patients most likely to benefit from cardioprotection (proximal occlusions with pre-PCI TIMI 0 flow).^5^ 995 patients were screened from 4 centers over a 2-year period but only 67 patients had CMR data. CMR during the acute setting was not possible at one of the recruiting centers but transport was provided for patients to go to another center (that was 1 and a half hours away) for the scan. However, this resulted in a large number of patients dropping out of the study following initial randomization. Furthermore, following the publication of the ALBATROSS trial^19^ and the study by van den Berg et al.,^15^ a decision was made by the trial steering committee to stop the study early due to slow recruitment and futility. Data on cardiac enzymes were not reported due to small sample size and a large number of missing data. However, MI size by CMR is considered more robust^26^ and was available for the majority of the patients in the study.

### Conclusion

This pilot study showed no benefit of intravenous MRA administered prior to reperfusion combined with 3 months of oral MRA therapy on reducing MI size in STEMI patients but there was an improvement in LV remodeling. Our pilot data might help to design further studies that are adequately powered to assess whether MRA therapy in STEMI patients without heart failure could reduce reperfusion injury and translate to an improvement in clinical outcomes.

## References

[1] Nabel E.G., Braunwald E. (2012). A tale of coronary artery disease and myocardial infarction. N Engl J Med.

[2] Ezekowitz J.A., Kaul P., Bakal J.A. (2009). Declining in-hospital mortality and increasing heart failure incidence in elderly patients with first myocardial infarction. J Am Coll Cardiol.

[3] Bulluck H., Foin N., Tan J.W. (2017). Invasive Assessment of the Coronary Microcirculation in Reperfused ST-Segment-Elevation Myocardial Infarction Patients: Where Do We Stand?. Circ Cardiovasc Interv.

[4] Frohlich G.M., Meier P., White S.K. (2013). Myocardial reperfusion injury: looking beyond primary PCI. Eur Heart J.

[5] Bulluck H., Yellon D.M., Hausenloy D.J. (2016). Reducing myocardial infarct size: challenges and future opportunities. Heart.

[6] Schmidt K., Tissier R., Ghaleh B. (2010). Cardioprotective effects of mineralocorticoid receptor antagonists at reperfusion. Eur Heart J.

[7] Bulluck H., Frohlich G.M., Mohdnazri S. (2015). Mineralocorticoid receptor antagonist pretreatment to MINIMISE reperfusion injury after ST-elevation myocardial infarction (the MINIMISE STEMI Trial): rationale and study design. Clin Cardiol.

[8] Beygui F., Vicaut E., Ecollan P. (2010). Rationale for an early aldosterone blockade in acute myocardial infarction and design of the ALBATROSS trial. Am Heart J.

[9] Bulluck H., Go Y.Y., Crimi G. (2017). Defining left ventricular remodeling following acute ST-segment elevation myocardial infarction using cardiovascular magnetic resonance. J Cardiovasc Magn Reson.

[10] Bulluck H., Rosmini S., Abdel-Gadir A. (2016). Impact of microvascular obstruction on semiautomated techniques for quantifying acute and chronic myocardial infarction by cardiovascular magnetic resonance. Open Heart.

[11] Bulluck H., Bryant J.A., Lim M.X. (2017). Full left ventricular coverage is essential for the accurate quantification of the area-at-risk by T1 and T2 mapping. Sci Rep.

[12] Goetti R., Kozerke S., Donati O.F. (2011). Acute, subacute, and chronic myocardial infarction: quantitative comparison of 2D and 3D late gadolinium enhancement MR imaging. Radiology.

[13] Yancy C.W., Jessup M., Bozkurt B. (2013). 2013 ACCF/AHA guideline for the management of heart failure: executive summary: a report of the American College of Cardiology Foundation/American Heart Association Task Force on practice guidelines. Circulation.

[14] Ibanez B., James S., Agewall S. (2018). 2017 ESC Guidelines for the management of acute myocardial infarction in patients presenting with ST-segment elevation: The Task Force for the management of acute myocardial infarction in patients presenting with ST-segment elevation of the European Society of Cardiology (ESC). Eur Heart J.

[15] van den Berg T.N.A., van Swieten H.A., Vos J.C., Verweij V., Wouterse A.C., Deinum J. (2016). Eplerenone does not limit ischemia-reperfusion injury in human myocardial tissue. Int J Cardiol.

[16] Li X., Qi Y., Li Y. (2013). Impact of mineralocorticoid receptor antagonists on changes in cardiac structure and function of left ventricular dysfunction: a meta-analysis of randomized controlled trials. Circ Heart Fail.

[17] Pitt B., Remme W., Zannad F. (2003). Eplerenone, a selective aldosterone blocker, in patients with left ventricular dysfunction after myocardial infarction. N Engl J Med.

[18] Montalescot G., Pitt B., Lopez de Sa E. (2014). Early eplerenone treatment in patients with acute ST-elevation myocardial infarction without heart failure: The Randomized Double-Blind Reminder Study. Eur Heart J.

[19] Beygui F., Cayla G., Roule V. (2016). Early Aldosterone Blockade in Acute Myocardial Infarction: The ALBATROSS Randomized Clinical Trial. J Am Coll Cardiol.

[20] Beygui F., Van Belle E., Ecollan P. (2018). Individual participant data analysis of two trials on aldosterone blockade in myocardial infarction. Heart.

[21] Bossard M., Binbraik Y., Beygui F. (2018). Mineralocorticoid receptor antagonists in patients with acute myocardial infarction - A systematic review and meta-analysis of randomized trials. Am Heart J.

[22] Dahal K., Hendrani A., Sharma S.P. (2018). Aldosterone antagonist therapy and mortality in patients with st-segment elevation myocardial infarction without heart failure: a systematic review and meta-analysis. JAMA Intern Med.

[23] Brilla C.G., Zhou G., Matsubara L. (1994). Collagen metabolism in cultured adult rat cardiac fibroblasts: response to angiotensin II and aldosterone. J Mol Cell Cardiol.

[24] Zannad F., Alla F., Dousset B. (2000). Limitation of excessive extracellular matrix turnover may contribute to survival benefit of spironolactone therapy in patients with congestive heart failure: insights from the randomized aldactone evaluation study (RALES). Rales Investigators. Circulation.

[25] Weir R.A., Mark P.B., Petrie C.J. (2009). Left ventricular remodeling after acute myocardial infarction: does eplerenone have an effect?. Am Heart J.

[26] Bulluck H., Hammond-Haley M., Weinmann S. (2017). Myocardial Infarct Size by CMR in Clinical Cardioprotection Studies: Insights From Randomized Controlled Trials. JACC Cardiovasc Imaging.

